# Perioperative Schmerztherapie bei minimal-invasiver Thoraxchirurgie

**DOI:** 10.1007/s00101-023-01329-6

**Published:** 2023-09-19

**Authors:** Katharina Bach, Christian Volberg, Thomas Wiesmann, Hinnerk Wulf, Ann-Kristin Schubert

**Affiliations:** 1 Klinik für Anästhesie und Intensivtherapie, Universitätsklinikum Marburg, Philipps-Universität Marburg, Baldingerstraße, 35043 Marburg, Deutschland; 2 Klinik für Anästhesiologie, operative Intensivmedizin und Schmerztherapie, Diakoneo Diak Klinikum Schwäbisch Hall, Schwäbisch Hall, Deutschland

**Keywords:** Thorakoskopie (VATS), Regionalanästhesie, PROSPECT, Brustwand, Analgesie, Thoracoscopy (VATS), Regional anesthesia, PROSPECT, Thoracic wall, Analgesia

## Abstract

Thorakale Eingriffe sind häufig mit starken postoperativen Schmerzen verbunden. Obwohl diese bei thorakoskopischem Vorgehen in der Regel weniger ausgeprägt sind, sorgen die intraoperative mechanische Irritation, Kompression oder Verletzung von Interkostalnerven wie auch die häufig eingelegten Thoraxdrainagen für therapiebedürftige Schmerzen. Eine adäquate Schmerztherapie ist in der Thoraxchirurgie essenziell, da eine insuffiziente Inspiration infolge unzureichender Schmerzkontrolle das Auftreten postoperativer Komplikationen fördert. Bei der Thorakotomie galt die Epiduralanästhesie lange als Goldstandard. Für die videoassistierte Thorakoskopie wird sie aufgrund von Nutzen-Risiko-Überlegungen teils nicht mehr empfohlen. Es existieren alternative Thoraxwandblockaden wie der Paravertebralblock, der Erector-spinae-plane-Block und der Serratus-anterior-plane-Block, für deren Einsatz die Studienlage teilweise heterogen ausfällt.

Dieser Artikel stellt die aktuellen Empfehlungen bezüglich des perioperativen Schmerzmanagements bei thorakoskopischen Eingriffen dar und gibt einen Überblick zu aktuellen PROSPECT-Empfehlungen sowie der aktuellen AWMF-Leitlinie zu peri- und postoperativer Schmerztherapie. Insbesondere werden einzelne regionalanästhesiologische Techniken und deren aktuelle Evidenz beleuchtet.

## Abstract (en)

Thoracic surgery is often associated with severe postoperative pain levels. Even though these are less pronounced in thoracoscopic approaches, mechanical irritation, compression or injury of intercostal nerves and placement of chest tubes can cause pain levels, which must be treated. An adequate pain therapy in thoracic surgery is essential as insufficient inspiration due to inadequate analgesia may result in postoperative complications. Epidural anesthesia was considered the gold standard in thoracotomy for a long time. For video-assisted thoracoscopy, however, it is sometimes no longer recommended due to its benefit-risk ratio. Alternative thoracic blocks are the paravertebral block, the erector spinae plane block and the serratus anterior block, for which research has found heterogeneous results.

This article summarizes the current recommendations for perioperative management of thoracoscopic surgery and gives an overview of the PROSPECT recommendations as well as the current Association of the Scientific Medical Societies in Germany (AWMF) guidelines for perioperative and postoperative pain therapy. In particular, individual regional anesthesia techniques and their current evidence are reviewed.

Thoraxchirurgische Eingriffe führen bei einem Großteil von Patient*innen postoperativ zu moderaten bis starken Schmerzen [21]. Eine insuffiziente Analgesie kann zu einer erhöhten Komplikationsrate führen. Ein suffizientes perioperatives Analgesiekonzept ist daher essenziell. Neuroaxiale oder periphere regionalanästhesiologische Verfahren stellen eine effektive Alternative und/oder sinnvolle Ergänzung in der perioperativen Schmerztherapie dar. Im Jahr 2022 sind zur Analgesie in der Thoraxchirurgie neue Empfehlungen herausgegeben worden.

1. Bei einem 45-jährigen Patienten (185 cm, 74 kg, ASA-Status II) ist eine linksseitige videoassistierte Thorakoskopie (VATS) bei persistierendem apikalem Spontanpneumothorax und Ausbildung einer Lungenfistel auf gleicher Seite geplant. Der Patient ist vorerkrankt mit einer normochromen, normozytären Anämie unklarer Genese. Zudem besteht ein Nikotinabusus mit 36 pack years. Die plasmatische Gerinnung ist normwertig. Es soll eine keilförmige Resektion des Segments I mit Entfernung des dystelektatischen Lungenparenchyms und der Emphysembullae durchgeführt werden.

## Einleitung

Im Jahr 2021 wurden in Deutschland fast 170.000 Operationen an Bronchus, Lunge, Pleura und Zwerchfell vorgenommen [30]. Ein Teil dieser Operationen bedarf nach wie vor der Versorgung mittels Thorakotomie, beispielsweise bei akuten, vital bedrohlichen Blutungen oder bei Tumorinfiltrationen an schwer erreichbaren Lokalisationen. Infolge des recht ausgeprägten Gewebetraumas der Thoraxwand sowie der potenziellen Gefahr von Rippenfrakturen und Nervenläsionen, verursacht durch das Spreizen der Rippen, sind Thorakotomien mit starkem postoperativem Schmerz vergesellschaftet. Dieser ist häufig Mitursache einer erhöhten postoperativen Morbidität und Mortalität. Durch die Reduktion ebenjenes operativen Traumas bietet ein minimal-invasives Vorgehen deutliche Vorteile hinsichtlich postoperativer Schmerzen und pulmonaler Komplikationen. So können nach thorakoskopischen Operationen eine frühere Mobilisation erreicht und damit auch die Patientenzufriedenheit und Lebensqualität verbessert werden [2]. Vorteile hieraus ergeben sich v. a. für ältere Patient*innen sowie Patient*innen mit mehr Vorerkrankungen und höherem ASA-Status [27, 36].

Durch den Fokus auf eine frühzeitige Mobilisation und kurze Hospitalisierungszeiten ist der Anteil der minimal-invasiven Thoraxchirurgie ständig zunehmend. Während die VATS initial vornehmlich zu diagnostischen Zwecken genutzt wurde, konnten in den letzten Jahren in vielen deutschen Kliniken mittels VATS erfolgreich minimal-invasive Operationsverfahren für Lungen‑, Mediastinal- und Ösophaguserkrankungen etabliert werden [4]. Ursache für den vermehrten Einsatz der VATS ist auch das umschriebenere Resektionsvorgehen. Während für die Lungenkrebschirurgie in den 1930er-Jahren lediglich die Pneumektomie zur Verfügung stand, wurde in den 1960er-Jahren die Lobektomie führend, die seither den Goldstandard darstellt. Vor dem Hintergrund besserer diagnostischer Bildgebung werden zunehmend Segment- oder Wedge-Resektionen erwogen [28].

In den letzten Jahren hat sich im klinischen Alltag ebenfalls die roboterassistierte Thorakoskopie (RATS) etabliert [25]. Aktuell ist sie der VATS wegen längerer Operationszeiten oft noch unterlegen, bietet jedoch die Möglichkeit zur 3D-Optik sowie flexibleren Instrumentenbewegung und damit zu potenziell besseren Präparationsmöglichkeiten [33].

Mithilfe von Neuerungen wie der Laserresektion von pulmonalen Metastasen können auch multiple Foci mittels VATS parenchymsparend reseziert werden [18].

Eine ebenfalls vielversprechende Weiterentwicklung, insbesondere für die operative Versorgung von schwer pulmonal oder kardial vorerkrankten Patient*innen, aber auch im Sinne der Fast-Track-Chirurgie bei Patient*innen mit einem ASA-Status von I und II, hat sich mit der Möglichkeit der nichtintubierten videoassistierten Thorakoskopie (NIVATS) aufgetan, die bereits in den 1950er-Jahren (vor der verbreiteten Nutzung von Doppellumentuben und der Einlungenventilation) beschrieben wurde. Bei der NIVATS wird der thorakale Eingriff an sedierten und regionalanästhesierten Patient*innen unter Erhaltung der Spontanatmung durchgeführt [32]. Sie findet zunehmend Anwendung bei Keilresektionen, Probeexzisionen oder Pleurodesen [12]. In einem systematischen Review mit Metaanalyse von 1684 Fällen verglichen Zhang et al. 2019 die NIVATS mit der VATS bei intubierten Patient*innen. Sie konnten ein niedrigeres Gesamtkomplikationsrisiko mit entsprechend niedrigerer Mortalität, kürzeren Operations- und Anästhesiezeiten, kürzeren Liegezeiten der Thoraxdrainagen, weniger Thoraxschmerz sowie insgesamt kürzerem Krankenhausaufenthalt in der Gruppe der NIVATS-Operationen feststellen [40]. Auch für die NIVATS können Thoraxwandblockaden eine Alternative zur EDA darstellen. In den ERAS-Empfehlungen wird die NIVATS als Verfahren mit Potenzial bezeichnet, jedoch wird davon abgeraten, sie als Routineverfahren zu nutzen [2]. Ziel der Enhanced-Recovery-After-Surgery(ERAS)-Konzepte sind die Verringerung postoperativer Komplikationen und die Beschleunigung der Genesung – und damit frühzeitige Entlassung – mithilfe eines multimodalen, multidisziplinären und evidenzbasierten Ansatzes.

(2) Präoperativ wird linksseitig ultraschallkontrolliert eine thorakale Paravertebralblockade (PVB) als einzeitiges Verfahren („single shot“) mit 15 ml 0,2 %iger Ropivacainlösung angelegt. Die Operation wird in Allgemeinanästhesie durchgeführt; eine Einlungenventilation wird mittels Bronchusblocker erreicht. Postoperativ wird der Patient extubiert sowie katecholaminfrei und mit geringfügigem Sauerstoffbedarf auf die IMC-Station verlegt.

### Konsequenzen eines thorakoskopischen Vorgehens für die perioperative Schmerztherapie

Bei der VATS liegt das Schmerzprofil, wie 2016 von Bendixen et al. gezeigt, zwar deutlich unter dem einer Thorakotomie [3], dennoch gab circa ein Drittel der Patient*innen in den ersten beiden Tagen nach der VATS ein moderates bis hohes Schmerzniveau an, das einer Intervention bedurfte. Insbesondere die mechanische Reizung, Kompression oder Verletzung von Interkostalnerven durch die Inzision, Rippenspreizer, Trokare oder Thoraxdrainagen gelten als Risikofaktoren für den verstärkten postoperativen Schmerz [13]. Der perioperativen Schmerztherapie kommt in der Thoraxchirurgie eine besondere Bedeutung zu. Ziel ist neben der subjektiven Erträglichkeit eine möglichst geringe Beeinträchtigung relevanter physiologischer Funktionen. Insbesondere eine erleichterte Atmung zur Vermeidung von Dystelektasen und Atelektasen sowie die Reduktion der Pneumonierate kann zu Patientensicherheit und Vermeidung postoperativer Komplikationen beitragen [2].

Für operative Eingriffe am Thorax stehen im Sinne eines multimodalen Analgesiekonzepts neben neuroaxialen Verfahren auch interfasziale Blockaden wie der Erector-spinae-plane(ESP)-Block, der Serratus-anterior-plane(SAP)-Block oder die Paravertebralblockade (PVB), genauso wie Interkostalblockaden und Wundrandinfiltrationen, zur Verfügung [24]. Die detaillierte Kenntnis von Art und Umfang der geplanten Operation ist unerlässlich. Durch enge interdisziplinäre Kommunikation können die perioperative Analgesie, das postoperative Outcome sowie die Patientenzufriedenheit optimiert werden.

### Regionalanästhesie zur Schmerztherapie nach VATS

Die aktuell gültige S3-Leitlinie der Arbeitsgemeinschaft der Wissenschaftlichen Medizinischen Fachgesellschaften e. V. (AWMF) „Behandlung akuter perioperativer und posttraumatischer Schmerzen“ ist 2022 erschienen. Sie stützt sich für den Abschnitt zur Schmerztherapie der Thoraxwand auf die PROSPECT-Empfehlung (s. unten) von 2015 zur Thorakotomie und unterscheidet entsprechend nicht zwischen Blockaden der Thoraxwand für konventionelle Thorakotomien oder VATS als zugrunde liegendem Eingriff. In dieser Leitlinie wird die EDA für thorakale Eingriffe (bei fehlender Kontraindikation) mit einer Kombination von Lokalanästhetikum und Opioid für 2 bis 3 Tage postoperativ (Empfehlungsgrad A) empfohlen. Die PVB wie auch der ESP-Block werden als Alternativen zur thorakalen EDA genannt [1]. Insbesondere Schmerzen beim Husten oder bei Mobilisation, die häufig durch einliegende Thoraxdrainagen provoziert werden, können mit einer EDA oder einer PVB suffizient therapiert werden [34, 39].

Das Akronym PROSPECT („*pro*cedure *spe*cific postoperative pain managemen*t*“) bezeichnet ein unabhängiges akademisches Gremium innerhalb der European Society of Regional Anaesthesia and Pain Therapy (ESRA), das sich v. a. aus Chirurg*innen und Anästhesist*innen zusammensetzt. Seit seiner Gründung 2002 veröffentlicht es, seinem Namen entsprechend, systematische Reviews zur Schmerztherapie für gängige chirurgische Eingriffe. Diese sollen die Therapie und Entscheidungsfindung im klinischen Alltag evidenzbasiert unterstützen. 2022 veröffentlichten Feray et al. ein systematisches Review mit spezifischen Empfehlungen für die Schmerztherapie nach VATS. Hier werden die PVB wie auch der ESP-Block als Erstlinientherapie und der SAP-Block als Zweitlinientherapie empfohlen. Die EDA wird gegenüber den neueren Thoraxwandblockaden explizit nicht mehr empfohlen. Begründet wird dies mit einem nachteiligen Komplikationsprofil bei fehlender Überlegenheit der analgetischen Potenz [10]. In 4 der berücksichtigten Studien war die thorakale EDA dem gegenübergestellten Verfahren (einmal Sufentanil-PCIA, 3‑mal PVB (sowohl als Single shot als auch als katheterbasiertes Verfahren)) zwar sehr wohl in der Schmerzlinderung überlegen. In 5 anderen Studien dagegen war die thorakale EDA gleichwertig oder unterlegen in Verbindung mit mehr Nebenwirkungen. Bei den verglichenen Verfahren handelte es sich einmal um chirurgisch angelegte Interkostalblockaden plus Ketorolac, Fentanyl-PCIA und Ketamin; in 3 Fällen um eine PVB und in einem Fall um eine extrapleurale Blockade. Hinsichtlich des Nebenwirkungsprofils traten in mehreren Studien Hypotensionen und Harnverhalte in der Gruppe mit thorakaler EDA auf. Die Studiengrundlage für eine Nichtempfehlung der thorakalen EDA darf somit als nicht vollkommen eindeutig gewertet werden. Eine Übersicht der PROSPECT-Empfehlungen ist in Tab. 1 dargestellt.

**Table 1:** 

	Empfohlen	Nicht empfohlen
Prä- und intraoperativ	Paracetamol NSAID/COX2-Inhibitoren Dexmedetomidin^a^	Gabapentinoide Kortikosteroide Lidocain i.v. Magnesiumsulfat Wundrandinfiltration Intrapleurale Infiltration Interkostalnervenblockade Transkutane elektrische Nervenstimulation (TENS)^b^
Postoperativ	Paracetamol NSAID/COX2-Inhibitoren Opioide (Rescue-Medikation)	Gabapentinoide Lidocain i.v. Dexmedetomidin TENS
Regionalanästhesie	Paravertebralblockade Erector-spinae-plane-Block Seratus-anterior-plane-Block	Thorakale Epiduralanästhesie

^a^Die ausschließlich intraoperative Gabe von Dexmedetomidin wird aufgrund von niedrigeren postoperativen Schmerzniveaus und weniger Agitation empfohlen, sofern es sich nicht um Patienten mit kardialem Risiko handelt

^b^Sämtliche genannten Maßnahmen sind einzig aufgrund von fehlender oder inkonsistenter Evidenzlage nicht empfohlen

## Regionalanästhesiologische Verfahren

Im Folgenden werden für die von PROSPECT und AWMF empfohlenen regionalanästhesiologischen Verfahren die Indikationen, Evidenz und Anlagetechniken orientierend beschrieben. Für weiterführende Informationen wird auf aktuelle Übersichtsartikel verwiesen [31]. Zu beachten ist bei allen drei dargestellten Blöcken die (im Gegensatz zur EDA) unilaterale Wirkung bei einseitiger Anlage. Eine tiefe viszerale Analgesie kann mit den meisten interfaszialen Blockaden nicht erreicht werden [5]. Eine Ausbreitung des Lokalanästhetikums bis hin zu den Spinalnerven sowie dem sympathischen Grenzstrang, durch die eine viszerale Analgesie erreicht wird, ist, abgesehen von den neuroaxialen Verfahren, nur mit der PVB möglich. Für ESP-Blockaden ist die Datenlage hierzu nicht eindeutig [5]. Tendenziell ist allenfalls von einer geringen Rate einer viszeralen Analgesie auszugehen.

Die Nähe zur Pleura ist bei allen regionalanästhesiologischen Techniken zu beachten, wobei eine akzidentelle Pleurapunktion bei einer anschließend geplanten VATS von geringer klinischer Relevanz für die Patient*innen ist.

Die PVB, der ESP- und der SAP-Block sind katheterbasiert möglich. In den der PROSPECT-Empfehlung zugrunde liegenden Studien waren es in der Regel katheterbasierte PVB, die mit der EDA verglichen wurden. Zwar bergen katheterbasierte Verfahren ein höheres Infektionsrisiko und das Risiko einer Dislokation im Verlauf, sind aber aufgrund der Möglichkeit der kontinuierlichen Schmerztherapie bei oft fortdauernder Reizung durch einliegende Thoraxdrainagen in vielen Fällen sinnvoller als ein einzeitiges Verfahren.

(3) Bei persistierendem Pneumothorax und prolongierter Einlage der Thoraxdrainage erhält der Patient am dritten postoperativen Tag bei mäßig kontrolliertem Dauerschmerz trotz oraler Schmerzmedikation sowie schmerzbedingt deutlich erschwerter Atemtherapie ultraschallkontrolliert einen Serratus-anterior-plane(SAP)-Block mit Anlage eines Katheters als kontinuierliches schmerztherapeutisches Verfahren. Über diesen werden nach Aufspritzen mit 20 ml 0,2 %iger Ropivacainlösung kontinuierlich 4 ml/h 0,2 %ige Ropivacainlösung verabreicht, und der Patient wird fortan 2‑mal täglich vom anästhesiologischen Akutschmerzdienst visitiert. Acht Tage nach der primären Operation erfolgt die thorakoskopische Revision bei persistierendem Pneumothorax mit operativer Blutpleurodese. Nach 3 weiteren Tagen kann die Thoraxdrainage entfernt und der Schmerzkatheter gezogen werden. Zwei Tage später wird der Patient bei guter Schmerzkompensation nach Hause entlassen.

### Gerinnungsmanagement

Für die genannten regionalanästhesiologischen Verfahren bestehen aktuell keine dezidierten Empfehlungen bezüglich hämostaseologischer Parameter und antithrombotischer Medikation. Während die Guidelines der *American Society of Regional Anaesthesia *(ASRA) von 2018 für tiefe Blockaden wie die PVB ein Gerinnungsmanagement entsprechend dem für eine EDA empfehlen, wird bezüglich anderer peripherer oder Plexusblockaden ein Vorgehen angepasst an Komprimierbarkeit, Vaskularisation und Blutungskonsequenzen des Punktionsareals vorgeschlagen [16]. Grundsätzlich sollten Risiken und Nutzen bei Vorliegen einer kompromittierten Gerinnung oder antithrombotischer Vormedikation individuell abgewogen werden.

Da bei regionalanästhesiologischen Verfahren oft höhere Mengen an Lokalanästhetika verwendet werden, muss auch das Risiko einer Lokalanästhetikaintoxikation als seltene, jedoch potenziell schwere iatrogene Komplikation berücksichtigt werden. Insbesondere die durch ihre Lipophilie lang wirksamen Präparate Ropivacain und Bupivacain weisen eine geringe therapeutische Breite in Bezug auf zentralnervöse und kardiale Toxizität bei der versehentlichen intravasalen Injektion auf. Bei intrapleuraler sowie interkostaler Applikation entstehen zudem die höchsten Plasmakonzentrationsanstiege [41]. Zur Prävention einer versehentlichen intravasalen Injektion sollte stets aspiriert und fraktioniert injiziert werden. Ein ultraschallgestütztes Vorgehen wird der landmarkengestützten Punktion vorgezogen. Da eine Hypoxie die Toxizität von Lokalanästhetika verstärkt, ist auf eine suffiziente Oxygenierung zu achten [38].

### Paravertebralblockade

Neben Brustwandeingriffen wie der Mastektomie sind thoraxchirurgische Indikationen für die PVB beispielsweise Rippenserienfrakturen, thorakoskopische Eingriffe oder Thorakotomien [31].

**Figure 1:**
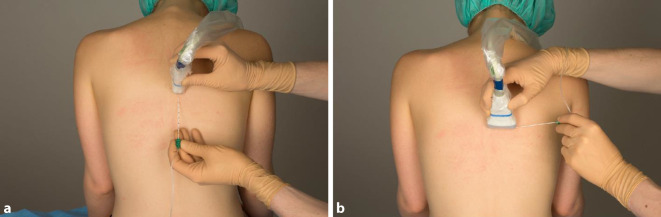

**Figure 2:**
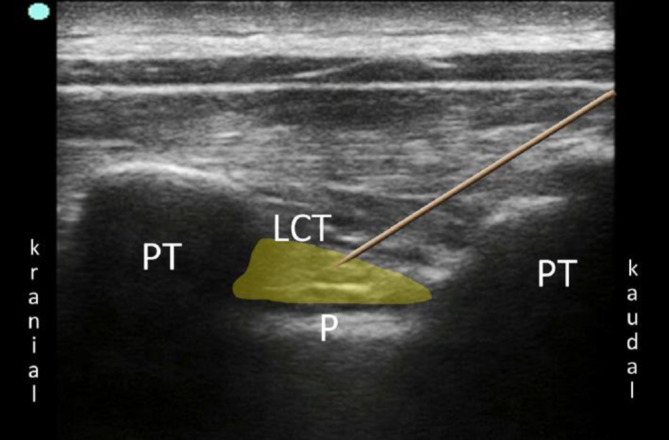

Bei der PVB wird ein Lokalanästhetikumdepot in den Paravertebralraum eingebracht. Hierzu werden Patient*innen in Seitenlage, Bauchlage oder eine sitzende Position gebracht. Sonographisch wird lateral des Processus spinosus zwischen Rippe und Querfortsatz die interne Interkostalmembran dargestellt. Dies kann in transversaler und sagittaler Schallausrichtung erfolgen, mit anschließender Punktion „in plane“ zum Schallkopf, entweder von lateral oder kranial kommend (Abb. 1). Bei korrekter Lage der Spitze der Punktionskanüle im Paravertebralraum tritt häufig ein Widerstandsverlust auf, und bei der Injektion weitet sich der Paravertebralraum mit konsekutiver Verschiebung der Pleura nach ventral (Abb. 2). Für einen Single shot oder das initiale Aufspritzen des Katheters können 15–20 ml 0,2- bis 0,5 %ige Ropivacainlösung verwendet werden; beim katheterbasierten Verfahren sollte die Laufrate maximal 5–6 ml/h betragen [31].

Da es sich bei der Zielstruktur um einen tiefliegenden Raum ohne Möglichkeit der Kompression handelt, muss mit Schwierigkeiten beim Beherrschen einer lokalen Blutung und einem Risiko für hämorrhagische Komplikationen gerechnet werden. Daher ist das notwendige Gerinnungsmanagement mit dem für eine EDA vergleichbar [16].

Die analgetische Potenz der PVB ist vergleichbar mit der einer EDA. Hingegen sind die Inzidenzen von Hypotension, Übelkeit und Harnverhalt geringer [7]. So ist die PVB – wie auch in den PROSPECT- und AWMF-Leitlinien geschehen – in der Nutzen-Risiko-Abwägung der EDA in der Regel vorzuziehen.

Das Gerinnungsmanagement für die PVB sollte dem für eine EDA entsprechen

### Erector-spinae-plane-Block

Der im Jahr 2016 erstmals beschriebene ESP-Block vereint die oben genannten Vorteile der PVB mit einer komplikationsärmeren Anlagetechnik bei dennoch suffizienter Analgesie [11].

Indikationen sind u. a. die Thorakotomie, VATS, das Postthorakotomie-Schmerzsyndrom sowie Rippenserienfrakturen [31].

**Figure 3:**
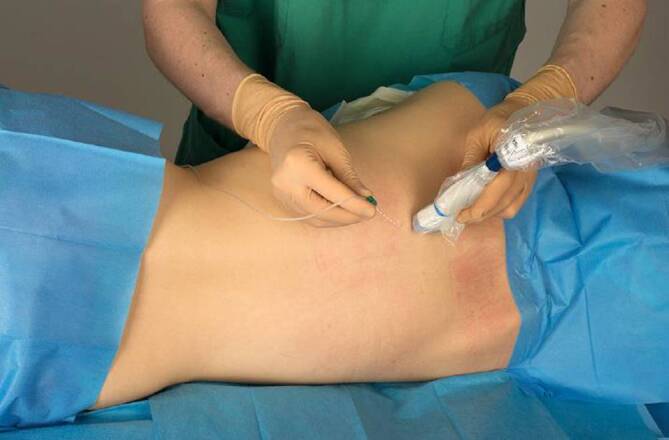

**Figure 4:**
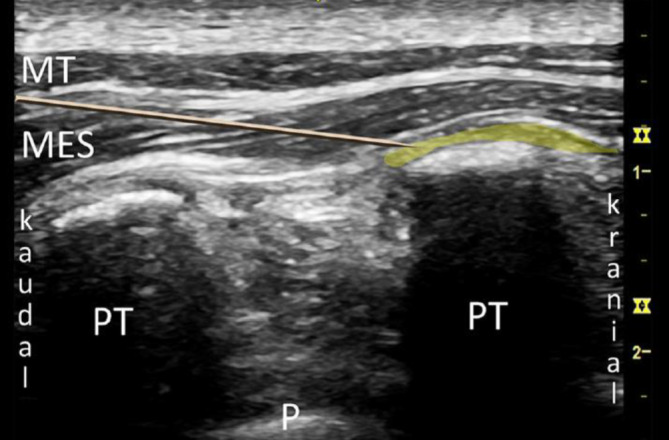

Für die Anlage werden Patient*innen in eine sitzende Position oder Seitenlage gebracht. Sonographisch wird in sagittaler Ausrichtung der Querfortsatz auf der gewünschten Höhe (für thoraxchirurgische Operationen in der Regel Th 5) unterhalb des M. erector spinae aufgesucht. Auf ebendiesen Querfortsatz wird von kranial in plane die Kanülenspitze vorgeschoben (Abb. 3). Bei Injektion wird der M. erector spinae vom Querfortsatz abgehoben, das Lokalanästhetikum breitet sich sagittal aus (Abb. 4). Für einen Single shot oder zum initialen Aufspritzen können je nach Quelle beispielsweise 20–40 ml 0,2- bis 0,5 %ige Ropivacain- oder 0,25 %ige Levobupivacainlösung verwendet werden [14, 31].

Für den Nutzen und die analgetische Potenz des ESP-Blocks in der Thoraxchirurgie gibt es vielversprechende kleinere Studien und Fallberichte [19]. Es wird weiterer vergleichender Studien bedürfen, um eine abschließende evidenzbasierte Aussage treffen zu können.

### Serratus-anterior-plane-Block

Thoraxchirurgische Indikationen für einen SAP-Block sind beispielsweise ventrale Rippenserienfrakturen [31].

**Figure 5:**
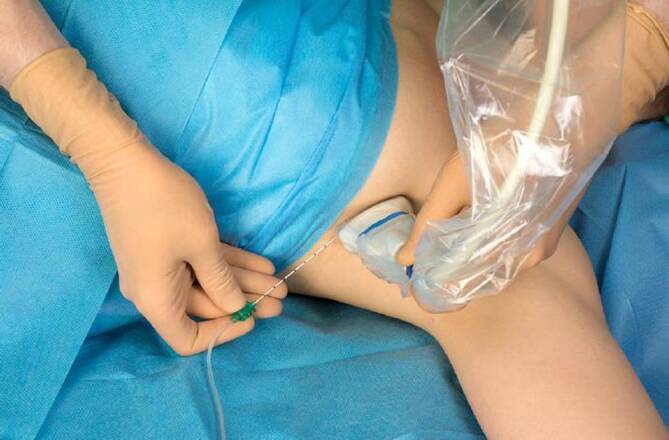

**Figure 6:**
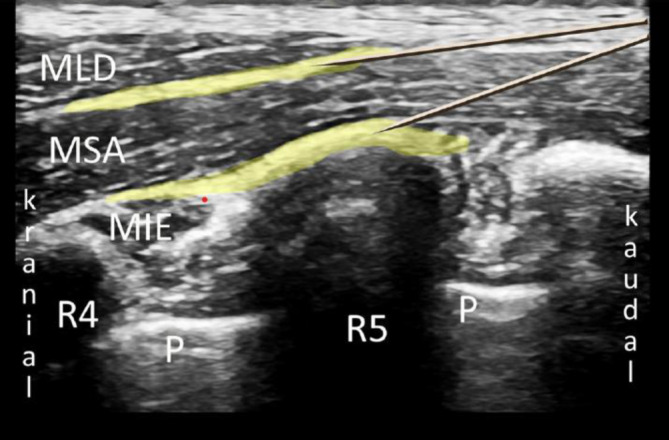

Für die Anlage wird in Rückenlage der Arm der zu infiltrierenden Seite abduziert. Sonographisch wird in der mittleren Axillarlinie in sagittaler Ausrichtung die Faszie des M. serratus anterior dargestellt. In plane kann von medial infiltriert werden (Abb. 5). Es werden ein oberflächlicher und tiefer SAP-Block unterschieden, bei denen das Lokalanästhetikum entweder ober- oder unterhalb des M. serratus anterior eingebracht wird (Abb. 6). Steinfeld et al. empfehlen die Verwendung von 20–40 ml 0,2- bis 0,5 %iger Ropivacainlösung [31]. Es existieren Fallberichte über Rippenfrakturen oder Thorakotomien, in denen eine Laufrate von 7 ml/h unter Verwendung von 0,0625- bis 0,1 %iger Bupivacainlösung angegeben wurde [20, 23].

Durch die Infiltration zwischen die Faszienblätter wird ein Ausschalten des N. intercostobrachialis sowie lateraler Äste der Interkostalnerven erreicht, was in der Regel eine Anästhesie der anterioren Brustwand auf Höhe Th 2–Th 6 sowie der lateralen Brustwand auf Höhe Th 2–Th 8 bewirkt [6].

Eine Metaanalyse von Liu et al. 2020 zeigte einen analgetischen Nutzen des SAP-Blocks nach thoraxchirurgischen Eingriffen [22]. Speziell für die VATS gibt es einzelne Studien, die auch hier einen Nutzen (für ein katheterbasiertes Verfahren) belegen [15].

### Interkostale Nervenblockade

Einzelne Blockaden, auch in Kombination mit einer Morphin-PCIA, sind der thorakalen EDA laut AWMF-Leitlinie unterlegen. Die Kombination aus 5 Interkostalblockaden und Morphin-PCIA ist der thorakalen EDA hingegen nur in den ersten 12 h postoperativ unterlegen, anschließend gleichen sich die NRS-Werte (Numerische Rating Skala zur subjektiven Einordnung von Schmerzen) an [1]. Gemäß der AWMF-Leitlinie kann eine interkostale Nervenblockade entsprechend zur Anwendung kommen, wenn eine PVB oder EDA nicht möglich ist. Hierfür können je 2–5 ml 0,2- bis 0,75 %ige Ropivacainlösung pro Injektionsstelle verwendet werden [31].

### Wundrandinfiltration

In der PROSPECT-Leitlinie beruht die Entscheidung gegen eine Empfehlung zur Wundrandinfiltration auf einer Studie, in der kein Unterschied im postoperativen Schmerzlevel zwischen Patient*innen mit und ohne Wundrandinfiltration gezeigt werden konnte [10].

Aufgrund der Einfachheit der Durchführung, der geringen Invasivität und des günstigen Nebenwirkungsprofils kann die Wundrandinfiltration bei Fehlen einer sonstigen neuroaxialen oder regionalanästhesiologischen Schmerztherapie eine Alternative darstellen. Aus diesen Gründen wird sie in der AWMF-Leitlinie für viele Eingriffe empfohlen, so auch für die Sternotomie in der Kardiochirurgie. Eine Stellungnahme zur (minimal-invasiven) Thoraxchirurgie existiert nicht [1].

### Zwischenfazit

In den meisten Studien, die den oben zitierten Leitlinien zugrunde liegen, werden die EDA sowie Thoraxwandblockaden gegenüber einer patientenkontrollierten intravenösen Analgesie (PCIA) getestet und sind dieser in der analgetischen Potenz praktisch nie überlegen. Im klinischen Alltag steht anstelle einer PCIA jedoch oft nur eine Analgesie auf Abruf zur Verfügung. Entsprechend ist kritisch zu hinterfragen, ob auch unter diesen Umständen ein adäquates analgetisches Niveau erreicht werden kann, oder ob die Indikation zur Anlage einer Regionalanästhesie nicht großzügiger gestellt werden sollte.

## Systemische Analgetikatherapie

Maßnahmen zur Regionalanästhesie nehmen in der perioperativen Medizin einen großen Stellenwert ein. Die Regionalanästhesie sollte im Sinne eines multimodalen Schmerzkonzepts mit einer systemischen Analgetikagabe kombiniert werden. Zudem ist gelegentlich die Anlage einer EDA oder eines anderen regionalanästhesiologischen Verfahrens zwar empfohlen, wird aber entweder von Patientenseite abgelehnt oder ist aus anderen Gründen kontraindiziert oder technisch nicht möglich. Im Folgenden wird eine Übersicht zur Therapie mit Opioiden (Opiate sollen hiermit gleichermaßen eingeschlossen sein) und Nichtopioidanalgetika (NOPA), inklusive alternativen analgetischen Verfahren, gegeben.

### Opioidanalgetika

In den ERAS-Empfehlungen zur Lungenchirurgie wird zu einer geringstmöglichen Anwendung von Opioiden geraten [2]. Die oben zitierte AWMF-Leitlinie stellt voran, dass eine orale Opioidgabe einer parenteralen Applikation – wann immer möglich – vorzuziehen ist. Außerdem sollen Opioide durch die Kombination mit NOPA eingespart werden [1]. In der AWMF-Leitlinie wird mit Hinweis auf fehlende Studien keine Dosisempfehlung für die orale Opioidtherapie gegeben. Tab. 2 listet eine Auswahl der oral verfügbaren Opioidanalgetika mit ihrer äquianalgetischen Potenz zu oralem Morphin sowie beispielhaften bzw. üblichen Startdosen für opioidnaive Patient*innen und der Maximaldosis über 24 h auf. Sofern eine i.v.-Opioidtherapie notwendig ist, wird die PCIA mit Bolusfunktion und Sperrintervall als Verfahren der Wahl genannt, da eine höhere Patientenzufriedenheit bei weniger Nebenwirkungen erreicht wird [17]. Die gebräuchlichen PCIA-Einstellungen für die Präparate Oxycodon, Piritramid, Hydromorphon sowie Morphin sind in Tab. 3 dargestellt. Besonderheiten hinsichtlich der Anwendung bei VATS existieren gegenüber anderen Indikationen nicht.

**Table 2:** 

Oral verfügbare Substanzen	Äquianalgetische Potenz	Startdosis (mg)	Intervall (h)	Maximaldosis (mg)
Tilidin (+ Naloxon)	0,1	50/4	12	600
Tramadol	0,1	50	12	400
Tapentadol	0,4	50	4–6	600
Morphin	1	10	12	
Oxycodon + Naloxon	5	5/2,5	12	160
Hydromorphon	7,5	4	12

**Table 3:** 

Parameter	Oxycodon	Piritramid	Hydromorphon	Morphin
Konzentration	1–2 mg/ml	1,5–3 mg/ml	0,2 mg/ml	1–2 mg/ml
Bolusgröße	1–2 mg	1,5–3 mg	0,2 mg	1–2 mg
Sperrzeit	10–15 min	10–15 min	10–15 min	5–15 min
Basalinfusion	–	–	–	–
Maximum	15 mg/4 h	15–25 mg/4 h	4 mg/4 h	10–20 mg/4 h

Ergänzend wird darauf hingewiesen, dass auch bei der PCIA als Verfahren der Akutschmerztherapie eine adäquate Schulung und Einweisung der Behandler*innen erfolgen sollte und strukturelle Voraussetzungen gegeben sein müssen, um potenzielle Risiken abzuwenden. Als Voraussetzungen werden genannt: eine sorgfältige Patientenauswahl, Standards zur Anwendung der PCIA und deren Monitoring, ein Akutschmerzdienst sowie gut geschultes Stationspersonal [9].

### Nichtopioidanalgetika

In der AWMF-Leitlinie werden die Aussagen zur medikamentösen Schmerztherapie nicht spezifisch für Thoraxeingriffe gemacht. Es gibt eine Grad-A-Empfehlung dafür, NOPA im Rahmen eines multimodalen Analgesieregimes zu verabreichen. Hierzu wird auf mehrere Cochrane-Analysen verwiesen, die für NSAID, Metamizol und Paracetamol einen schmerzreduzierenden Effekt sowie die Einsparung von Opioiden zeigen konnten. Während die Analysen häufig auf Einmalgaben beruhen, wird darauf hingewiesen, NOPA als Basisanalgetika in festen Zeitintervallen zu verabreichen, die sich nach der jeweiligen Wirkdauer einer Substanz richten sollten [1]. Eine komprimierte Übersicht ist in Tab. 4 wiedergegeben:

**Table 4:** 

Präparat	Verabreichung	Dosisintervalle/Tag	Max. Tagesdosis	Besonderheiten
Diclofenac	p.o.	2- bis 3‑mal 50 mg Retard: ein- bis 2‑mal 75 mg	150 mg	Nierenzellschädigung Magenulkus/obere gastrointestinale Blutung^a^ Wechselwirkung von Ibuprofen mit ASS
Ibuprofen	p.o. i.v.	2- bis 3‑mal 200–800 mg 2- bis 3‑mal 400–600 mg	2400 mg	
Metamizol	p.o./i.v.	3- bis 4‑mal 500–1000 mg	4000 mg	Hypotonie bei schneller Infusion Allergie, Agranulozytose^b^
Paracetamol	p.o./i.v.	3- bis 4‑mal 500–1000 mg	4000 mg	Leberzellschädigung^c^
Parecoxib	i.v.	2- bis 4‑mal 20–40 mg	80 mg	Verabreichung für max. 3 Tage

^a^Insbesondere bei längerer Anwendung von NSAR wie Ibuprofen sollte auf einen zusätzlichen Magenschutz, beispielsweise mit Protonenpumpeninhibitoren, geachtet werden, um gastrointestinale Nebenwirkungen zu minimieren

^b^Gemäß der AWMF-Leitlinie sollten Patient*innen über die Symptome einer Agranulozytose aufgeklärt und die Nachbehandler*innen über die Anwendung von Metamizol informiert werden

^c^In der AWMF-Leitlinie wird darauf hingewiesen, dass besonders fastende und kachektische Patient*innen sowie Patient*innen mit Alkoholabusus oder anderweitig vorbestehendem Leberschaden ein erhöhtes Risiko für eine (zusätzliche) Leberschädigung infolge Paracetamoltherapie haben

Da Paracetamol gegenüber NSAID, COX-2-Hemmern und Metamizol weniger wirksam ist, sollen laut einer weiteren Grad-A-Empfehlung letztere bei der Applikation bevorzugt werden [1]. In der ERAS-Empfehlung wird Paracetamol aufgrund seines geringen Kontraindikations- und Nebenwirkungsprofils als wichtiger Teil der postoperativen Schmerztherapie hervorgehoben [2]. Bei PROSPECT wird darauf verwiesen, dass die Evidenzlage für die Kombination von Paracetamol, NSAID und/oder COX‑2 Hemmern für andere Eingriffe gut ist, sodass trotz fehlender Evidenz für die VATS im Speziellen eine Empfehlung ausgesprochen wird [10].

Als Option wird in der AWMF-Leitlinie eine Kombination aus niedrig dosiertem Ibuprofen und Paracetamol (beispielsweise Ibuprofen 200 mg plus Paracetamol 500 mg) genannt, die auch in der ERAS-Empfehlung Erwähnung findet [1, 2].

Bezüglich unerwünschter Arzneimittelwirkungen (UAW) von NOPA (gastrointestinale Blutungen, Nieren- oder Leberfunktionsstörungen, kardiovaskuläre Ereignisse, Knochenmarkdepression etc.) wird in der AWMF-Leitlinie auf Cochrane-Analysen verwiesen, die für einen Behandlungszeitraum von 1 bis 5 Tagen bei Patient*innen ohne Komorbiditäten keine erhöhte Inzidenz für UAW fanden. Demgegenüber wird auf einen kritischen Einsatz bei vorliegenden Kontraindikationen hingewiesen. In der Leitlinie werden für ausgewählte, gebräuchliche Präparate Wechselwirkungen und Kontraindikationen genauer aufgelistet [1].

Da Metamizol im angloamerikanischen Raum sowie in vielen europäischen Ländern nicht zugelassen ist, findet es in der ERAS- und PROSPECT-Empfehlung keine Erwähnung. Nach Meinung der Autor*innen kann aber ein NSAID mit Metamizol vermutlich ähnliche additive Effekte zeigen wie oben für Paracetamol und Ibuprofen beschrieben. Daten hierzu fehlen im Wesentlichen.

### Gabapentinoide

Für neuropathischen Schmerz stellen Gabapentinoide Medikamente der ersten Wahl dar [29].

Gabapentinoide werden in der PROSPECT-Empfehlung für die thorakoskopische Chirurgie aufgrund der inkonsistenten Evidenz nicht empfohlen. Dabei konnte in den zugrunde liegenden Studien durchaus eine Reduktion des postoperativen Schmerzniveaus gezeigt werden, und auch die Inzidenz von neuropathischem Schmerz war signifikant geringer [10].

Auch in der AWMF-Leitlinie wird der allgemeine routinemäßige Einsatz von Gabapentinoiden nicht empfohlen. Begründet wird dies mit einer Risiko-Nutzen-Abwägung: Bei einem klinisch oft nicht relevanten Einfluss auf das postoperative Schmerzniveau haben Gabapentinoide ein recht ausgeprägtes Nebenwirkungsprofil (z. B. Risiko für Schwindel, Sehstörungen und Abhängigkeit, Akkumulation von Pregabalin bei Nierenfunktionsstörung). Der Einsatz von Gabapentin wäre postoperativ zudem als Off-Label Use zu werten. Für diverse Eingriffe (lap. Cholezystektomie und Sleeve-Gastrektomie) wird allerdings zur Verabreichung von Gabapentinoiden geraten, sofern die Gabe der üblichen Basisanalgetika nicht möglich ist [1].

Da eine neuropathische Schmerzkomponente infolge einer Reizung der Interkostalnerven auch nach thorakoskopischen Eingriffen nicht auszuschließen ist, können Gabapentinoide dennoch einen Stellenwert haben.

Üblicherweise wird Gabapentin 3‑mal täglich mit 100 mg/Gabe und Pregabalin 2‑mal täglich mit 25 mg begonnen und sukzessive gesteigert [35].

### Lidocain per continuitatem

Außer in den Empfehlungen für die konventionelle Prostatektomie findet dieses Analgesieverfahren bei anderen PROSPECT-Leitlinien keine weitere Erwähnung. In der AWMF-Leitlinie wird die i.v.-Lidocaingabe unabhängig vom Eingriff nicht empfohlen, jedoch für den Einzelfall als Reserveoption vorbehalten. In dem zugrunde liegenden systematischen Review von Weibel et al. 2018 konnte nach Auswertung von 68 randomisierten kontrollierten Studien kein klarer klinischer Nutzen von Lidocain für die Reduktion von Schmerzen oder den Opioidbedarf nachgewiesen werden – weder im Vergleich zu einem Placebo noch zur thorakalen EDA. Als Grund werden Ungenauigkeiten und Inkonsistenzen im Design der einzelnen untersuchten Studien genannt [37].

Sofern Lidocain per continuitatem dennoch erwogen wird, wird in der AWMF-Leitlinie folgende Dosierung empfohlen:

- 1,5 mg/kgKG Lidocainbolus vor Schnitt nach Narkoseeinleitung,
- intraoperative Lidocaininfusion von 1,5 mg/kgKG und h,
- postoperative Weiterführung für maximal 24 h bei 1,33 mg/kgKG und h [1].

Unerwünschte Wirkungen auf das Zentralnervensystem sowie das Herz-Kreislauf-System sind zu bedenken – insbesondere, wenn aufgrund von parallel eingesetzten regionalanästhesiologischen Verfahren die empfohlenen Maximaldosierungen überschritten werden. Zu beachten ist, dass die kontinuierliche i.v.-Gabe von Lidocain zur Schmerztherapie einen Off-Label Use darstellt, der einer gesonderten Aufklärung bedarf.

In der persönlichen Erfahrung der Autor*innen stellt die kontinuierliche i.v.-Lidocaingabe weniger eine zusätzliche Therapiemaßnahme als mehr eine sinnvolle Alternative bei Versagen eines neuroaxialen oder regionalanästhesiologischen Verfahrens für die minimal-invasive Thoraxchirurgie dar.

### Dexamethason

Eine Empfehlung für die intraoperative Gabe von – je nach Studie 4–10 mg – Dexamethason i.v. zu analgetischen und antiemetischen Zwecken findet sich in jeder PROSPECT-Leitlinie zur laparoskopischen Chirurgie und analog auch in der AWMF-Leitlinie. In der PROSPECT-Leitlinie zur VATS wird zwar Stellung zur präoperativen Gabe von 125 mg Methylprednisolon bezogen (und hier als ohne nennenswerten analgetischen Effekt bei jedoch erhöhten Blutglucosewerten beschrieben). Studien über die Gabe von Dexamethason bei VATS wurden jedoch nicht inkludiert [10]. Entsprechend wird die intraoperative Gabe von Dexamethason in der Thoraxchirurgie nicht empfohlen. Auch die AWMF-Leitlinie erwähnt die intraoperative Dexamethasongabe für die Thoraxchirurgie nicht.

Die antiemetische Wirkung von Dexamethason ist hingegen gut belegt [8]. In den ERAS-Empfehlungen wird einer Gabe von 8 mg präoperativ zur PONV-Prophylaxe eine Wirkung bis zu 72 h zugesprochen. Trotz potenzieller Erhöhung der Blutzuckerspiegel und postoperativer Infektionen war die Verabreichung von Dexamethason nicht mit einer erhöhten Komplikationsrate nach thoraxchirurgischen Eingriffen vergesellschaftet [2]. Ebenfalls gut belegt ist die perineurale oder i.v.-Injektion von Dexamethason zur Wirkverlängerung einer Regionalanästhesie und zur möglichen Einsparung von Opioiden [26]. Bei der Applikation von Dexamethason zur PONV-Prophylaxe kann man sich somit eine mögliche koanalgetische Wirkung zunutze machen.

### Transkutane elektrische Nervenstimulation

Wie auch Gabapentinoide wird die transkutane elektrische Nervenstimulation (TENS) in der PROSPECT-Empfehlung für die thorakoskopische Chirurgie aufgrund der inkonsistenten Evidenz nicht empfohlen [10].

Vorteile der TENS sind geringe Kosten, die einfache Anwendung, geringe Invasivität, das günstige Nebenwirkungsprofil (Hautrötung) und die patientenkontrollierte Anwendung, sodass die Empfehlungslage in der AWMF-Leitlinie aufgrund des guten Nutzen-Risiko-Profils zugunsten der Anwendung von TENS ausfällt. So wird es als zusätzliche Maßnahme „bei bestimmten Indikationen“ empfohlen. Für die Anwendung von TENS in der Thoraxchirurgie gibt es keine Stellungnahme [1].

### Ketamin

Gemäß AWMF-Leitlinie sollte „bei Patient*innen mit mittleren bis größeren operativen Eingriffen/einem hohen Risiko für starke oder anhaltende postoperative Schmerzen […] im Rahmen einer multimodalen/balancierten Analgesie perioperativ Ketamin verabreicht werden“. Hierzu zählen beispielsweise Patient*innen mit vorbestehender Opioideinnahme. Ergänzend wird auf das Suchtpotenzial hingewiesen [1]. Zwar wird Ketamin im Abschnitt zur Thoraxchirurgie nicht explizit hervorgehoben, dennoch werden Patient*innen, die sich einer VATS unterziehen, durchaus von den oben zitierten Kriterien erfasst.

Dosierungsvorschlag gemäß AWMF-Leitlinie:

- Bolus von ca. 0,5 mg/kgKG Ketaminracemat oder 0,25 mg/kgKG S(+)-Ketamin,
- anschließende Infusion von 0,5–0,75 mg/kgKG und h Ketaminracemat bis 45 min vor dem Operationsende oder 0,4–0,6 mg/kgKG und h S(+)-Ketamin bis 30 min vor dem Operationsende [1].

In der PROSPECT-Leitlinie wurden keine Studien zu Ketamin nach VATS berücksichtigt, sodass hierzu keine Empfehlung ausgesprochen wird [10].

## Fazit für die Praxis

- Die hochwirksame Epiduralanästhesie verliert aufgrund suffizienter, risikoärmerer regionalanästhesiologischer Alternativen in der minimal-invasiven Thoraxchirurgie an Stellenwert.
- Empfohlen werden
  - die Paravertebralblockade, eine tiefe Blockade mit eigenem Risikoprofil (Verletzung der Pleura, Pneumothorax, schwere Beherrschbarkeit von Blutungskomplikationen);
  - der Erector-spinae-plane-Block, dem trotz der oberflächlichen Anlage und damit günstigerem Risikoprofil das Erreichen einer viszeralen Analgesie zugesprochen wird.
- Andere oberflächliche Rumpfwandblockaden erreichen keine viszerale Analgesie, weshalb sie für die Behandlung von Schmerzen durch einliegende Thoraxdrainagen nach einer VATS eher unzureichend sind.
- Nichtopioidanalgetika sollten unter Berücksichtigung von Kontraindikationen und Wechselwirkungen angewendet werden.
- Opioide sollten zurückhaltend eingesetzt werden, bevorzugt oral oder als PCIA.
